# Biofabrication of SDF-1 Functionalized 3D-Printed Cell-Free Scaffolds for Bone Tissue Regeneration

**DOI:** 10.3390/ijms21062175

**Published:** 2020-03-21

**Authors:** Alina Lauer, Philipp Wolf, Dorothea Mehler, Hermann Götz, Mehmet Rüzgar, Andreas Baranowski, Dirk Henrich, Pol Maria Rommens, Ulrike Ritz

**Affiliations:** 1 Department of Orthopaedics and Traumatology, BiomaTiCS, University Medical Center, Johannes Gutenberg University, 55131 Mainz, Germany; laueralina@rocketmail.com (A.L.); phwolf70@gmail.com (P.W.); dorothea.mehler@unimedizin-mainz.de (D.M.); ruezgar1969@gmail.com (M.R.); andreas.baranowski@unimedizin-mainz.de (A.B.); pol.rommens@unimedizin-mainz.de (P.M.R.); 2 CBU—Cell Biology Unit, PKZI, University Medical Center, BiomaTiCS, Johannes Gutenberg University, 55131 Mainz, Germany; hgoetz@uni-mainz.de; 3 Department of Trauma, Hand and Reconstructive Surgery, Goethe University Frankfurt, 60590 Frankfurt am Main, Germany; d.henrich@trauma.uni-frankfurt.de

**Keywords:** bone tissue regeneration, 3D printed cell-free scaffold, polylactide, collagen type I, stromal-derived factor 1, in vivo model of critical size defects

## Abstract

Large segmental bone defects occurring after trauma, bone tumors, infections or revision surgeries are a challenge for surgeons. The aim of our study was to develop a new biomaterial utilizing simple and cheap 3D-printing techniques. A porous polylactide (PLA) cylinder was printed and functionalized with stromal-derived factor 1 (SDF-1) or bone morphogenetic protein 7 (BMP-7) immobilized in collagen type I. Biomechanical testing proved biomechanical stability and the scaffolds were implanted into a 6 mm critical size defect in rat femur. Bone growth was observed via x-ray and after 8 weeks, bone regeneration was analyzed with µCT and histological staining methods. Development of non-unions was detected in the control group with no implant. Implantation of PLA cylinder alone resulted in a slight but not significant osteoconductive effect, which was more pronounced in the group where the PLA cylinder was loaded with collagen type I. Addition of SDF-1 resulted in an osteoinductive effect, with stronger new bone formation. BMP-7 treatment showed the most distinct effect on bone regeneration. However, histological analyses revealed that newly formed bone in the BMP-7 group displayed a holey structure. Our results confirm the osteoinductive character of this 3D-biofabricated cell-free new biomaterial and raise new options for its application in bone tissue regeneration.

## 1. Introduction

The overall risk of fractures resulting in non-unions lies between 2% and 30% depending on age, gender, type and site of fracture, soft tissue damage and secondary illnesses (e.g., diabetes). In particular, large segmental bone defects occurring after trauma, resection of bone tumors, debridement of infections and/or revision surgeries can result in non-unions [1,2]. The resulting pain and limitations in terms of activities of daily life that these patients are immense, and there is also economic harm. Costs for tibia non-unions are doubled when compared to those without a non-union [3]. Although much knowledge was acquired during the last years for reconstruction of bone defects, e.g., employing new methods such as reaming irrigation aspiration (RIA) or concerning management of infected non-unions [4,5], the gold standard in therapy is still autologous bone grafting. This therapy requires additional interventions and, consequently, is combined with the risk of surgical complications and morbidity at the donor site. Moreover, the bone stock is limited [6]. Consequently, there is a high demand for new therapies capable of treating large segmental bone defects, which has led to great interest in bone tissue engineering. Different biodegradable and biocompatible materials employing various fabrication techniques have been developed and tested [7]. However, the optimal material fulfilling all clinical and mechanical requirements for a bone substitute in large diaphyseal defects still has to be found [8].

The 3D-printing techniques evolved in the last 20 years, leading to new optional materials for bone restoration. Currently, 3D printing, or 3D bioprinting, incorporating cells, extracellular matrix or bioactive molecules allows the fabrication of scaffolds with high structural complexity including pores of various sizes [9]. This relative new technique has already been applied in many fields of medicine including bone or cartilage restoration in dentistry or orthopedic surgery. The fabricated materials can be used as scaffolds for tissue regeneration, as prosthetic implants and/or as drug carriers [10]. 

Implan Table 3D-printed materials used as bone substitutes have to fulfill specific requirements: they need to be biocompatible, induce cell adhesion, proliferation and differentiation, be osteoconductive and, if possible, osteoinductive, demonstrate mechanical stability and be degradable with non-cytotoxic degradation products. Moreover, they should imitate extracellular matrix and it should be possible to either integrate cells or immobilize cytokines or growth factors. Many printable materials are available that can be used as bone substitute material, but the balancing act between stability, biocompatibility and degradation as well as mimicking the natural stiffness of bone is especially difficult. One solution could be composite materials combining stable structures with high stiffness and biomechanical stability with softer materials where bioactive molecules or cells can be included. Polymer filaments such as polylactide acid (PLA), polylactide (PDLLA), polycaprolactone (PCL), polypropylene fumarate (PPF), and polyether ether ketone (PEEK) can be used as hard biocompatible materials. They can be printed with fused deposition modeling (FDM) printers, which are cheap and can be handled as desktop printers. The materials melt at a temperature of approximately 200 °C, are pressed through a print head and can then be printed individually. These hard materials can be combined with soft materials such as hydrogels made from natural polymers such as collagen, polysaccharides, cellulose, gelatin, and alginate hyaluronic acid. Most of these materials are bioactive and can be modified with growth factors and/or loaded with cells of various sources [10].

One of the most utilized polymers for 3D printing is PLA. It is biocompatible, biodegradable, non-toxic and due to its melting temperature, at approximately 175 °C, it can be easily applied for FDM printing [11]. PLA allows cell adhesion but is not bioactive itself [12,13]. One major concern in applying PLA is long-term biocompatibility, as during degradation, acidic products might occur and decrease the physiological pH. However, surface modifications change the degradation product pattern [14]. Therefore, PLA is often combined with other materials [13]. One advantage of PLA is its good mechanical stability, although its compressive strength is lower when compared to bone tissue. PLA can easily be combined with soft and bioactive materials. Many options exist, but collagen as an extracellular matrix protein is one of the most widely used soft materials in tissue engineering [15,16]. It is inexpensive, biocompatible and degraded in the body by collagenases releasing non-toxic and non-immunogenic peptides [17]. Collagen can easily be loaded with bioactive molecules [18]. Moreover, it has been used in composite materials, combining hard materials such as strontium containing glass particles and soft materials [19]. Cells can be incorporated as demonstrated by Lee et al., who used collagen as bioink supplemented with pre-osteoblasts [20]. Among others, we printed PLA and collagen to induce tissue regeneration in bone defects [21]. Martin et al. printed PLA and functionalized the scaffolds with collagen, minocycline and citrate hydroxyapatite nanoparticles [22]. They could show that these scaffolds stimulated adhesion, proliferation and osteogenesis-related gene expression of hMSCs. Dewey et al. fabricated a multi-scale mineralized collagen PLA composite, which promoted osteogenic differentiation of porcine adipose stem cells [23]. Teixeira et al. evaluated the BMSC response to PLA scaffolds modified with polydopamine and type I collagen [24]. All these studies demonstrate the potential of PLA and collagen in combination as bone substitute material.

Bone morphogenetic proteins (BMP) are a group of growth factors belonging to the transforming growth factor-beta superfamily. They demonstrate osteogenic properties and induce bone regeneration and fracture healing [25]. It has been demonstrated that BMP-7 is completely absent in non-unions [26], whereas it is present in physiological bone, suggesting its application as therapy for fracture healing or after appearance of non-unions. BMP-7 was tested in many studies, with different results, and partly approved by the Food and Drug Administration (FDA) and the European Medicines Agency (EMA) [27,28]. Some severe side effects such as heterotopic ossification, osteolysis and inflammation were described, and a reevaluation of its clinical use is currently taking place. One important aspect is the concentration administered, as extreme high doses were used in some studies without testing different concentrations [25,29]. BMP-7 has been used in 3D printing with PCL and beta-tricalcium-phosphate. These scaffolds were tested in a pig condyle defect model and showed good tissue ingrowth and bone regeneration [30].

Another promising factor to be incorporated in scaffolds to induce bone regeneration is the stromal-derived factor 1 (SDF-1). It demonstrates various biological functions such as regulating cell migration and cell growth. By recruiting endothelial progenitor cells from the bone marrow, SDF-1 demonstrates a crucial role in angiogenesis [31]. Concerning bone regeneration, it stimulates homing of bone marrow mesenchymal stem cells [32]. It can be incorporated in hydrogels and keep its functionality [33].

In our former in vitro study [21], we printed solid discs as well as three-dimensional porous cages such as structures of polylactide with an inexpensive desktop printer and coated or filled them with type I collagen. We showed that various different cells adhere and proliferate on the solid discs. We immobilized SDF-1 in the three-dimensional PLA–collagen composite and demonstrated an even release for 48 h. After 48 h, 50% of the immobilized amount remained in the scaffold. We also demonstrated the functionality of the released growth factor. The study also confirmed the biocompatibility of our composite as well as endotoxin contamination clearly below the FDA limit.

In this study, we performed the proof of concept by employing a femur defect model in the rat. Rat is a standard model to analyze bone regeneration. As recommended by Garcia et al., we created a non-union using a large segmental defect model without periosteal or endosteal injuries [34]. We used a femur defect with a critical size defect of 6 mm in rats 10 weeks of age. This represents a segmental defect which was stabilized with a fixation device (PEEK plate; Research Implant System, AO Foundation, Davos, Switzerland) and filled with our created bone substitute material. Together with mice, rats are the standard model to evaluate new materials for bone regeneration and to study bone physiology [35,36]. Their skeletons and bone biology including cells are similar to humans [37]. A defect size of 6 mm in rat femora represents a critical size defect as already described by others [38,39,40].

We printed a PLA cylinder corresponding in size and diameter to the defect set in the rat femur. We filled the cylinder with type I collagen, immobilized SDF-1 or BMP-7 into the collagen, implanted the composite material into the rat femur defect and analyzed bone regeneration after 8 weeks with µCT and histological analyzes.

## 2. Results and Discussion

### 2.1. Biomechanical Testing

**Figure 1:**
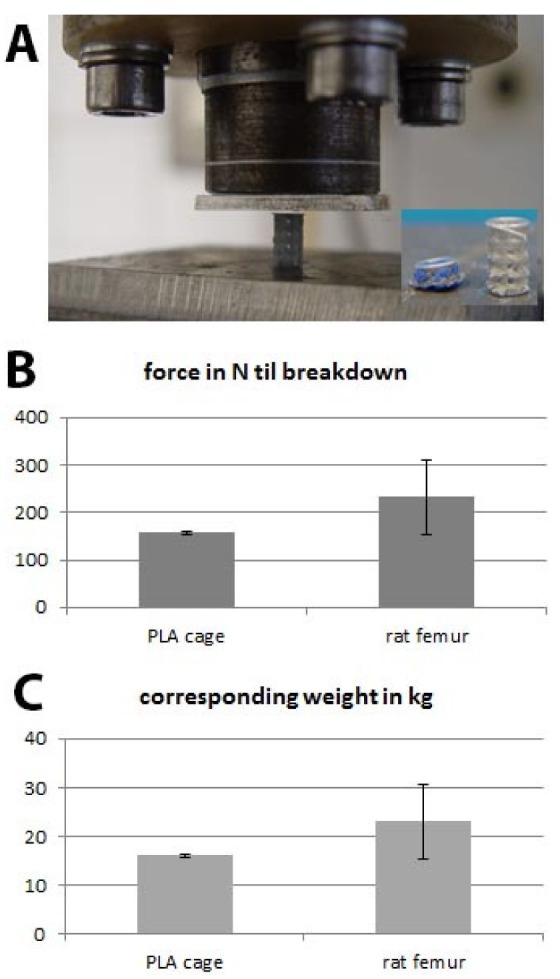
Biomechanical testing; (**A**) experimental setting; (**B**) applied force until breakdown of the polylactide acid (PLA) cage and rat femur and the (**C**) corresponding weight in kg.

Before the cylinders were implanted into rat femur osteotomy, they were tested for their mechanical stability using a pneumatic universal testing machine. Forces from 10 N to 500 N were applied with a frequency of 0.02 Hz until breakdown of the PLA cylinder (Figure 1A). The force applied until breakdown was 246 N +/− 77 N for rat femora and 156 N +/− 2 N for PLA cylinders. These values correspond to a bearing weight of 16 kg in the case of PLA cylinder and 24 kg concerning rat femur (Figure 1B,C).

The results show that the rat femur is more stable than 3D-printed polylactide. This was not surprising as it is known that PLA tends to brittle and demonstrates lower compressive strength compared to natural bone [10,41]. We decided that the mechanical stability of the PLA cylinder when combined with collagen would be sufficient to be used as bone substitute in the rat femur osteotomy especially as in our model the defect is stabilized with a PEEK plate and six screws.

### 2.2. Course of Bone Regeneration

**Figure 2:**
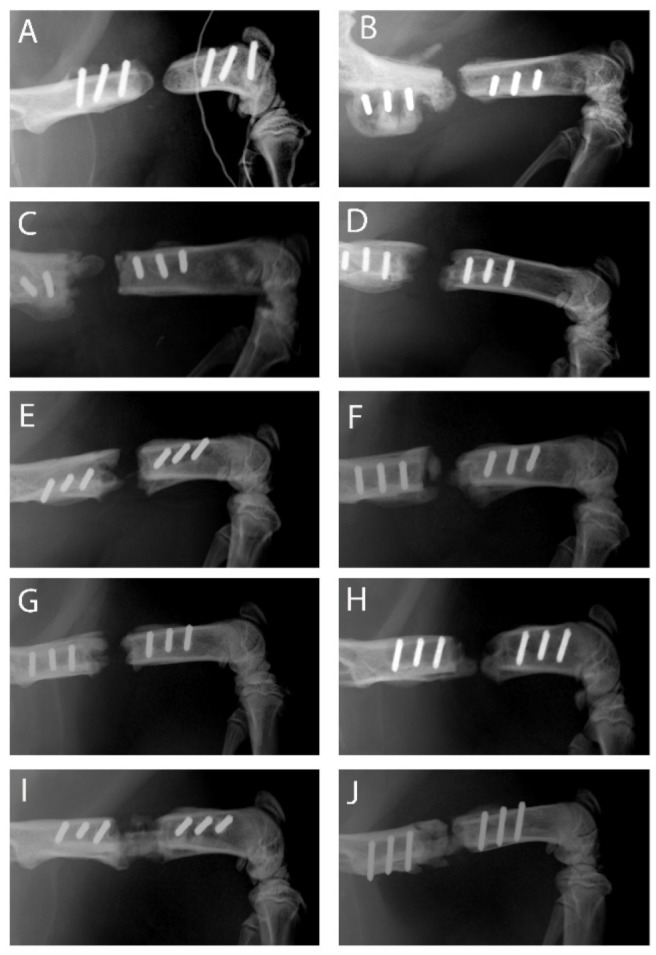
X-ray images of the different groups 4 weeks after surgery. Group 1—no implant—(**A**,**B**); 2—PLA cylinder alone—(**C**,**D**); 3—PLA cylinder + collagen type I—(**E**,**F**); 4—PLA cylinder + collagen type I + SDF-1—(**G**,**H**); 5—PLA cylinder + collagen type I + BMP-7—(**I**,**J**).

**Figure 3:**
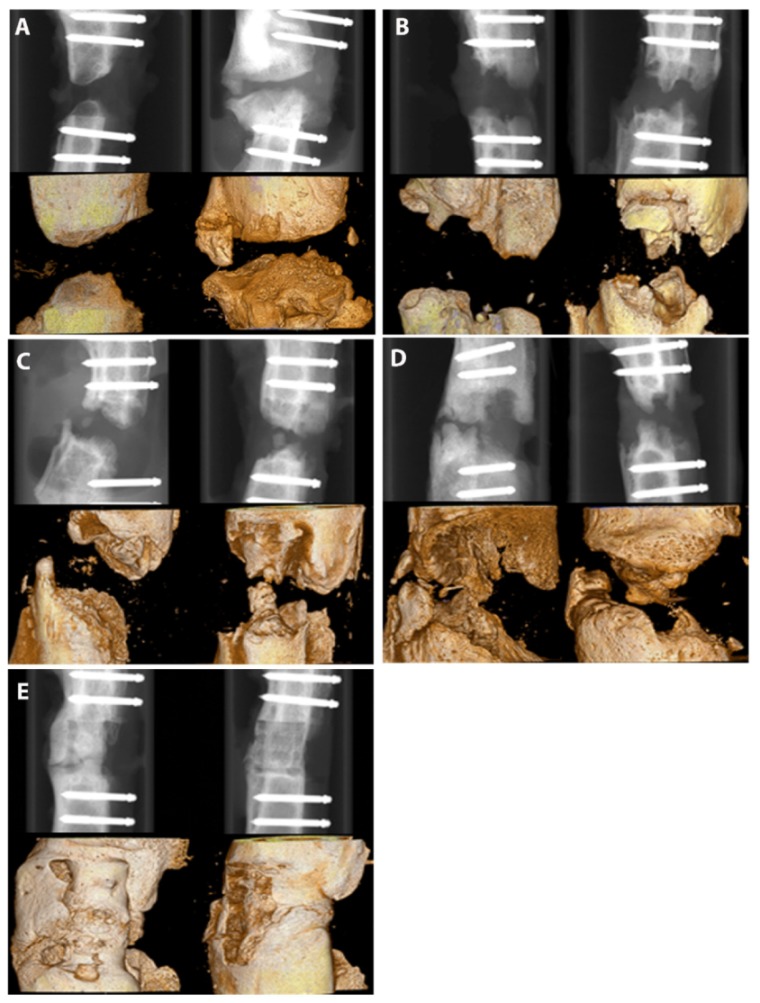
X-ray images of the different groups 8 weeks after surgery. Group 1—no implant—(**A**); 2—PLA cylinder alone—(**B**); 3—PLA cylinder + collagen type I—(**C**); 4—PLA cylinder + collagen type I + SDF-1—(**D**); 5—PLA cylinder + collagen type I + BMP-7—(**E**).

Rats were divided into five experimental groups (1: no implant; 2: PLA cylinder alone; 3: PLA cylinder + type I collagen; 4: PLA cylinder + type I collagen + SDF-1; 5: PLA cylinder + type I collagen + BMP-7) and operated as described in Methods. X-rays performed four weeks after surgery revealed heterogeneous results between the different groups. Observing the radiographs, one has to consider that neither the cylinder itself nor the stabilizing PEEK plate is visible. As shown in Figure 2, hardly any bone growth could be detected in groups 1 (no implant; Figure 2A,B), 2 (PLA cylinder alone; Figure 2C,D) and 3 (PLA cylinder + collagen type I; Figure 2E,F). In group 4 (PLA cylinder + collagen type I + SDF-1; Figure 2G,H), marginal bone growth at the edges of the defect can be detected. In group 5 (PLA cylinder + collagen type I + BMP-7; Figure 2I,J), bone growth through the cylinder was visible.

It is known that BMP-7 can induce bone regeneration already at a relative early time point [42]. Shi et al. observed an effect of SDF-1 already after 4 weeks [43], whereas only marginal bone regeneration could be observed after 4 weeks in our study. This different effect could be due to the material used. In contrast to our study, Shi et al. used demineralized bone matrix scaffolds as carrier systems, whereas we immobilized SDF-1 in collagen type I inside a PLA cylinder. These different materials and immobilization methods could be responsible for differences concerning the release and functionality of this chemokine.

Eight weeks after surgery, the animals were killed and x-rayed and the femora were excised for analyses employing µCT. As shown in Figure 3, more differences between the groups can be observed. In the group with no implant, development of non-unions is observed with the typical non-union formation described by Weber and Czech. No bridging of the fracture gap was seen (Figure 3A). In the group with PLA cylinder alone, a low osteoconductive effect can be observed (Figure 3B), which is more pronounced in the group where the cylinder is loaded with collagen type I (Figure 3C). In the group PLA+collagen+SDF-1, the fracture gap is almost closed, indicating a good osteoinductive effect of SDF-1 (Figure 3D). As already seen in the x-rays after 4 weeks, BMP-7 shows the most distinct effect on bone regeneration (Figure 3E).

**Figure 4:**
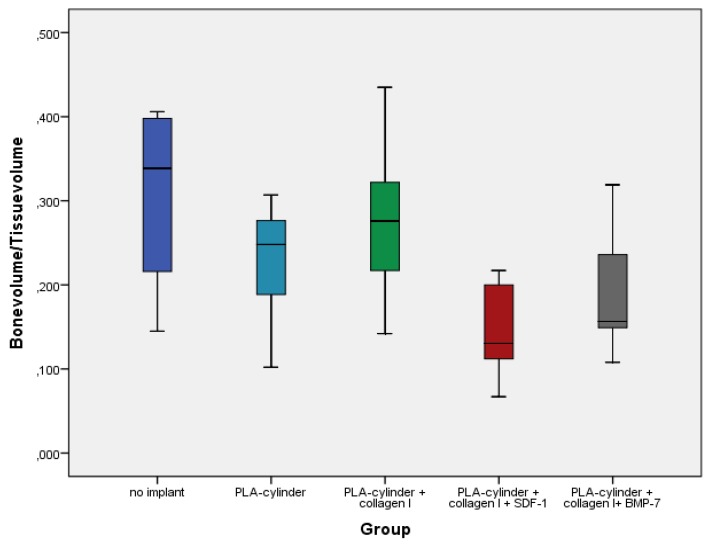
Quantitative analyses of bone volume/tissue volume ratios in the different groups 8 weeks after surgery.

Quantitative analyses of bone volume to total volume ratio revealed partly opposite results (Figure 4). The best ratios were observed in the groups without implant and in the PLA group without growth factors.

Although these results seem to be surprising at first glance, they can be easily explained. First, we quantified overall bone formation. In particular, in groups with no growth factors, we observe non-physiological and non-directed bone formation, which we measure with the ratio of bone volume to toal volume (BV/TV). Therefore, altogether, bone formation seems to be higher than in the groups with growth factors, where we observe directed bone formation aimed to close the fracture gap. The bone forms a callus, widening to the sides instead of growing straight through the fracture gap to close it, which is typical for complete or incomplete non-unions [44]. Second, the bone formed in the group without implant is a rather avital bone, probably consisting mostly of calcium-phosphate and much denser than the vital bone measured in the growth factor groups. These results illustrate that in this case, the quantitative analyses should be interpreted with caution and only in combination with imaging techniques such as µCT, X-ray and histology.

### 2.3. Histological Analyses

**Figure 5:**
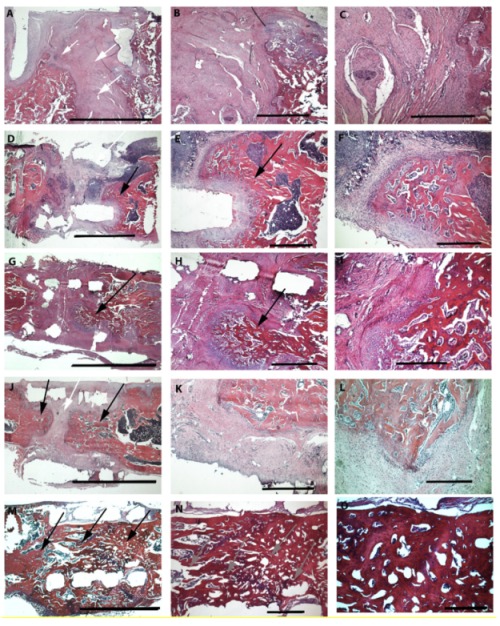
HE staining of various groups (8 weeks after surgery) under three different magnifications. First column overview—scale bar 5 mm; second column—30 times magnification—scale bar 1 mm; third column—75 times magnification—scale bar 500 µm. Group 1—no implant—(**A**–**C**); 2—PLA cylinder alone—(**D**–**F**); 3—PLA cylinder + collagen type I—(**G**–**I**); 4—PLA cylinder + collagen type I + SDF-1—(**J**–**L**); 5—PLA cylinder + collagen type I + BMP-7—(**M**–**O**). White arrows indicate regions with connective tissue, black arrows indicate bone growth, and grey arrows show holey structures of the bone tissue.

Histological analyses confirmed the qualitative µCT and X-ray results. In the groups with no implant or PLA cylinder alone, connective tissue dominates (Figure 5A, white arrows) and only marginal newly formed bone is observed. In the PLA group, slightly more bone tissue can be seen compared to the group without implant, especially in the areas between old bone and cylinder (Figure 5D,E, black arrows). In the group PLA + collagen type I, bone growth can be observed into the cylinder (Figure 5G,H, black arrows). In the group PLA + collagen type I + SDF-1, bone growth can be detected almost completely through the cylinder (Figure 5J, black arrows) and only the central part consists of pure connective tissue (Figure 5J, white arrows). In the group modified with BMP-7, bone growth can be observed completely through the cylinder (Figure 5M, black arrows). However, regarding the bone structure, there are tremendous differences between the bone formed after SDF-1 and BMP-7 supplementation. Whereas the new built bone in the SDF-1 group looks like the physiological bone, the bony structure in the BMP-7 group is holey (Figure 5N, grey arrows).

**Figure 6:**
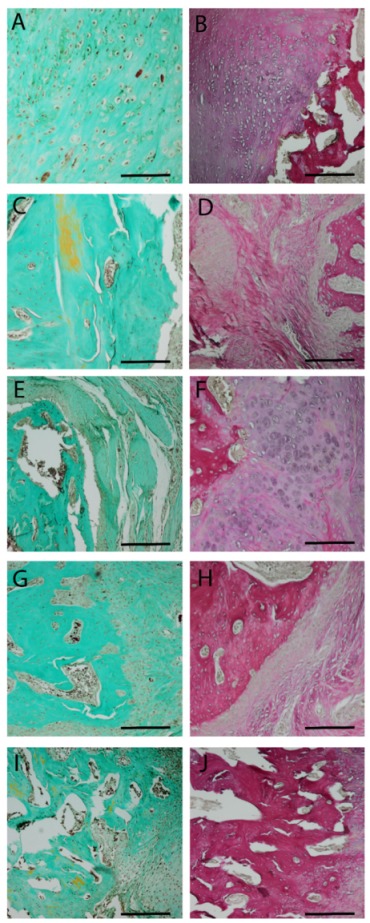
Masson–Goldner (first column) and Elastica van Gieson (second column) staining 8 weeks after surgery. Central parts of the fracture are shown. Group 1—no implant—(**A**,**B**); 2—PLA cylinder alone—(**C**,**D**); 3—PLA cylinder + collagen type I—(**E**,**F**); 4—PLA cylinder + collagen type I + SDF-1—(**G**,**H**); 5—PLA cylinder + collagen type I + BMP-7—(**I**,**J**). Scale bars: 1000 µm.

This impression is confirmed by Masson–Goldner trichrome staining (Figure 6). Whereas only light green colors, identifying connective tissue, can be observed in the groups with no implant (A) and PLA cylinder alone (C), a mixture of green-turquoise staining, representing bone, and connective tissue can be observed in the group with collagen (E). The amount of green-turquoise increases in the SDF-1 group (G) and is most present in the BMP-7 group (I). Again, the structure of the BMP-7 bone is holey and very porous. In order to observe differences in the formation of elastic fibers, muscle and small vessels an Elastica van Gieson staining was performed. No differences concerning elastic fibers or small vessel formation could be observed between the groups.

It has been demonstrated by us and others that administration of BMP-7 results in excessive bone formation [45], heterotopic ossification [46] and bone with an abnormal structure and diminished biomechanical properties [47]. This is confirmed by some other studies employing BMP-2. For example, Zara et al. demonstrated that high doses of BMP-2 induced formation of structurally abnormal bone and inflammation in vivo [48].

Therefore, we conclude that, although bone growth after administration of SDF-1 results in a slower progress of bone regeneration, the bone seems to be more physiological than after BMP-7 utilization. This might be due to the fact that SDF-1 promotes bone regeneration via recruitment of endogenous bone marrow-derived stem cells [43,49,50] and cell migration [51]. By loading PLA and collagen with SDF-1, the required cells for bone regeneration are homed. The usage of collagen as a carrier system for SDF-1 has also been shown for bone regeneration in dentistry as well as in in vivo applications. Further, other materials have been demonstrated to carry SDF-1 [52,53], which speaks for the good functionality of SDF-1 after release from carrier systems. These facts show its potential as a component to induce bone regeneration and for its use in TE with cell-free bone substitutes. One option might be to combine SDF-1 and BMP-7, resulting in a combination of faster bone growth in a physiological manner. Tan et al. and others demonstrated a good effect on bone regeneration when combining SDF-1 and BMP-2 in vitro and in vivo [33,54]. The combination of SDF-1 and BMP-7 still has to be explored and is part of further studies.

As described above, combinations of collagen type I or other polymers with SDF-1 show promising results concerning new biomaterials for tissue engineering. However, these materials often demonstrate too low a stability to be used as bone substitute. Therefore, our approach to include these materials in a mechanically stable cylinder of 3D-printed polylactide combines many advantages. Polylactide is a biocompatible material and it can be modulated with simple 3D printers individually and as requested. Polylactide can be loaded with collagen type I with immobilized SDF-1, which is released in a semi-controlled manner and the released SDF-1 is functional. To our knowledge, in addition to our approach, only two research teams worked with a combination of polylactide and SDF-1. He et al. [55] combined SDF-1 with hydrogels containing different ratios of polylactide and Damanik et al. [56] combined electrospun PLA scaffolds with SDF-1 and demonstrated a significantly higher infiltration of MSC.

In order to identify differences in the presence of elastic fibers and blood vessels in the different groups, we performed Elastica van Gieson (EVG) staining. No differences concerning elastic fibers can be observed between the groups. This is probably due to the late time point of our histological studies at 8 weeks. Differences would be observed after shorter time periods (3–4 weeks) as described by others [57,58,59]

In our setting, parts of the implanted polylactide are still present 8 weeks after implantation. Histological staining did not reveal inflammatory or cytotoxic reactions at this time. Although some studies report long-term cytotoxic effects (see introduction), many other studies confirm the biocompatibility of PLA [60,61,62] as a bone substitute or for fixation of bone fractures. We believe that the degradation of polylactide is very slow and that the body is able to degrade the resulting degradation products. The degradation characteristic of the PLA–collagen implant material will be tested in a long-term setting, with standing times up to six months after implantation. In this follow-up study, biomechanical testing will be performed after explantation to analyze the differences in the stability of the built bone in the various groups.

### 2.4. Limitation of the Study

One limitation of the study is that we did not perform quantitative analyses of our histological data as, in our opinion, the qualitative images sufficiently demonstrate the differences between the groups.

## 3. Materials and Methods

### 3.1. Scaffold Preparation

**Figure 7:**
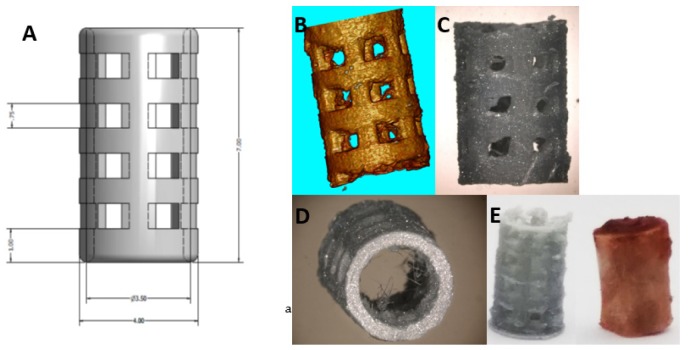
The 3D-printed PLA cylinder. SEM images (**A**–**D**) and printed PLA cage corresponding to an excised piece of rat femur (**E**).

A cylinder of PLA filament (Ultimaker silver metallic PLA, iGo3D, Hannover, Germany) with a diameter of 4 mm and a height of 7 mm was printed with the Ultimaker 2+. Mechanical, thermal and other properties are listed in the technical data sheet from Ultimaker. The cylinder was designed with a 3D modelling software (Autodesk Inventor Professional 2013, Autodesk, San Rafael, CA, USA) comparable to the size of the excised piece of femur during surgery (Figure 7E). Pores with a diameter of approximately 1 mm were included into the wall of the scaffold to facilitate bone ingrowth (Figure 7A–D). A collagen solution (10× M199, 6% NaHCO_3_, 2.5%, 65% collagen solution (Viscofan; 5 mg/mL 16.5% water) was prepared. In total, 100 µl of this solution was supplemented with 100 ng SDF-1 or BMP-7 (both Miltenyi Biotec, Bergisch Gladbach, Germany), respectively, and pipetted into the cylinder and polymerized at room temperature.

### 3.2. Biomechanical Testing

Before implantation, mechanical testing was performed using a pneumatic universal testing machine (SincoTec^®^, Clausthal-Zellerfeld, Germany). The settings for the testing machine were selected with the help of the control software PneuSys^®^. Vertical movements, defined as the difference between the starting versus the ending point of the force transmitting bar, were measured directly at the actuator of the testing machine being connected to the bar. The data were recorded by means of a data-logging card at a scanning rate of 200 Hz. The data were processed with a program written in Dasylab^®^ (DASYLab^©^ National Instruments Ireland Resources Limited, Provider: measX GmbH & Co.KG, Mönchengladbach, Germany). In order to reduce the data volume for post-processing, the arithmetic mean was calculated during the test for each twenty measurement values. This reduced data set was then read out in the Excel^®^ program for further post-processing. Independent measurements were performed for three PLA cylinders and three rat femora. The axial compression test was performed with a preload of 10N and a linear load increase to failure (or set force) at a frequency of 0.02 and 0.03 Hz.

### 3.3. In Vivo Model

Our study was approved by the local regional welfare committee (Landesuntersuchungsamt Rheinland-Pfalz AZ 23177-07/g17-1-032). All animal experiments complied with the ARRIVE (Animal Research: Reporting of In Vivo Experiments) guidelines and were carried out in accordance with the EU Directive 2010/63/EU for animal experiments. Animals were housed 2 per cage in the translational animal research center (TARC, University Medical Center, Mainz, Germany) with a 12 h dark–light rhythm.

In total, 36 ten-week-old Wistar rats (Janvier, France) were acclimatized for 3 days before they were subdivided into five groups according to Table 1:

To create the desired osteotomy of 6 mm, we used the rat fix system (RISystem, Davos, Switzerland). Anesthesia was initiated with isoflurane-oxygen per inhalation. Rats were anesthetized with an intra-peritoneal injection of midazolam (0.15 mg/kg), medetomidin (2 mg/kg) and fentanyl (0.005 mg/kg). As a pain prophylaxis, drinking water was supplied with tramadol (1 mg/mL) from 2 days before until 7 days after surgery. Skin incision was made along the femur, from the hip to the knee, and the underlying muscles were lifted. A gigly wire saw was placed around the bone and a PEEK plate was fixed with 6 screws bicortically. After positioning the drill and saw guide, a 6 mm femur osteotomy was created by using the gigly saw. The PLA cylinder was, if necessary, adapted in size and set in the resulted defect without further fixation. A previous pilot study for establishing the surgery method had demonstrated that this was the best option. The wound was then closed with resorbable vicryl sutures 4-0 (Ethilon. Ethicon, Norderstedt, Germany)).

X-rays were performed after 4 and 8 weeks. The rats were sacrificed 8 weeks after surgery by exposure to CO_2_. Femora were placed in 4.5% paraformaldehyde solution for µCT and histological analyses.

### 3.4. µCT Analyses/Bone Volume/Total Volume

Bone formation was evaluated using a high-resolution micro computed tomography (CT) scanner (CT 40, SCANCO Medical AG, Brüttisellen, Switzerland). Radiographs of the femur were performed with the following specifications: the specimen tube of the CT scanner containing the femur was run with an X-ray tube of 70 kV and 113 µA and the operational resolution was 30 µm. Per rotation, 1000 pictures were taken with 1200 × 1200 pixel resolution. Reconstruction resulted in 520 pictures/sample with a voxel size of 30 µm corresponding to a layer thickness of 30 µm and height of 6 mm. Thereby, the complete femur could be reconstructed.

The generated graphical material was exported as common DICOM images. It was edited and analyzed with the open source software “ImageJ” including the plugins “BoneJ” and “Stack Alignment.” The volume fraction (BV/TV) was then computed by measuring the bone volume (BV) inside a defined cylindrical total volume (TV). The base of the cylinder had a diameter of 5 mm and the cylinder had a height of 6 mm. This cylinder was orthogonally projected onto the bone defect and the calculation was performed using a voxel-based algorithm by the application “volume fraction” integrated in the BoneJ plugin.

### 3.5. Histology

After radiologic analysis, the femora were decalcified using a 10% EDTA solution for at least 6 weeks, with the solution exchanged every second day during the first two weeks and then weekly. The femora were dehydrated by the Sakura VIP E150 Tissue Processor (Sakura Finetek GmbH, Rüsselsheim, Germany) and then embedded in paraffin wax. The resulting blocks were cut in 5 µm slices, deparaffinized and then stained with hematoxylin and eosin.

The histologic slides were evaluated by descriptive histology by two independent and blinded investigators. The periphery and center of the critical size defects were compared among the five groups. Specimens were described and evaluated for cellular presence, extent of ossification defect and thickness of regenerated bone.

### 3.6. Masson–Goldner Trichrome Staining

Nuclei were stained for 5 min with hematoxylin according to Weigert, washed with 1% acetic acid followed by Ponceau-S staining (Sigma-Aldrich, Darmstadt, Germany) for 10 min. After another washing step with acetic acid (1%), samples were placed in acid orange G solution (Carl Roth, Karlsruhe, Germany) for 10 min. After rinsing in 1% acetic acid, samples were stained with light green (Merck Chemicals GmbH, Darmstadt, Germany) for 10 min then rinsed again in 1% acetic acid for 30 s.

### 3.7. Elastica Van Gieson Staining

Nuclei were stained for 5 min with hematoxylin according to Weigert then rinsed with distilled water for 10 min. After staining with resorcinol-fuchsine solution (Waldeck GmbH & Co. KG, Mnster, Germany), samples were again rinsed with distilled water for 10 min. Then samples were incubated with van Gieson solution (picric acid fuchsine; Waldeck GmbH & Co. KG, Münster, Germany) for 5 min, dehydrated in alcohols, cleared and mounted.

### 3.8. Statistical Analyses

For statistical analyses SPSS version 23.0 was used. Normal distribution was confirmed with Shapiro–Wilk test. Significant levels were analyzed with ANOVA and Tukey tests.

## 4. Conclusions

The 3D-biofabricated cell-free biomaterial consisting of polylactide in combination with collagen type I and especially when modified with SDF-1 is capable of inducing bone regeneration in a critical size defect in rats. In contrast to other biomaterials, this biomaterial can be easily produced with a simple 3D desktop printer. However, further experiments combining SDF-1 with bone morphogenetic proteins as well as large animal studies have to be performed to further characterize this material in terms of its capabilities in various applications.

## Figures and Tables

**Table 1:** Classification of groups.

Group		*n*
1	No implant	4
2	PLA cylinder	8
3	PLA cylinder+ collagen I	8
4	PLA cylinder + collagen I + SDF-1	8
5	PLA cylinder + collagen I + BMP-7	8
